# Pneumonia caused by *Bordetella bronchiseptica* in two HIV-positive patients: Pneumonia causada por *Bordetella bronchiseptica* em dois pacientes HIV-positivos

**DOI:** 10.1590/1516-3180.2015.02492701

**Published:** 2016-05-13

**Authors:** Roberta Filipini Rampelotto, Andreas Hörner, Christine Hörner, Roselene Righi, Rosmari Hörner

**Affiliations:** I MSc. Doctoral Student, Postgraduate Pharmaceutical Sciences Program, Universidade Federal de Santa Maria (UFSM), Santa Maria, RS, Brazil.; II Undergraduate Student, Universidade Federal de Santa Maria (UFSM), Santa Maria, RS, Brazil.; III Undergraduate Student, Universidade do Extremo Sul Catarinense (UNESC), Criciúma, SC, Brazil.; IV MSc. Pharmacist, Bacteriology Laboratory, Hospital Universitário de Santa Maria (HUSM), Universidade Federal de Santa Maria (UFSM), Santa Maria, RS, Brazil.; V PhD. Associate Professor, Department of Clinical and Toxicological Analyses, Universidade Federal de Santa Maria (UFSM), Santa Maria, RS, Brazil.

**Keywords:** Bordetella bronchiseptica, HIV, Pneumonia, Case reports [publication type], Humans

## Abstract

**CONTEXT AND OBJECTIVE:**

*Bordetella bronchiseptica* (BB) is a Gram-negative coccobacillus responsible for respiratory diseases in dogs, cats and rabbits. Reports on its development in humans are rare. However, in immunosuppressed patients, especially in those with the immunodeficiency virus (HIV), BB can cause severe pulmonary infections. We report on two cases of pneumonia caused by BB in HIV-positive male patients in a university hospital.

**CASE REPORT:**

The first case comprised a 43-year-old patient who was admitted presenting chronic leg pain and coughing, with suspected pneumonia. BB was isolated from sputum culture and was successfully treated with trimethoprim/sulfamethoxazole in association with levofloxacin. The second case comprised a 49-year-old patient who was admitted presenting fever, nausea, sweating and a dry cough, also with suspected pneumonia. BB was isolated from sputum culture, tracheal secretions and bronchoalveolar lavage. The disease was treated with ciprofloxacin but the patient died.

**CONCLUSION:**

BB should be included in the etiology of pneumonia in immunodeficient HIV patients. As far as we know, these two were the first cases of pneumonia due to BB to occur in this university hospital.

## RESUMO

**CONTEXTO E OBJETIVO:**

*Bordetella bronchiseptica* (BB) é um cocobacilo Gram-negativo responsável por causar doenças no trato respiratório de cães, gatos e coelhos. São raros os relatos do desenvolvimento desse microrganismo em seres humanos. Porém, em pacientes imunodeprimidos, especialmente nos portadores do vírus da imunodeficiência humana (HIV), a BB pode causar infecções pulmonares graves. Nós relatamos dois casos de pneumonia por BB em pacientes do sexo masculino, HIV-positivos em um hospital universitário.

**RELATO DE CASO:**

No primeiro caso, o paciente de 43 anos foi internado apresentando dor crônica nos membros inferiores e tosse com suspeita de pneumonia. Na cultura de escarro, foi isolado BB, e a infecção foi tratada com sucesso com a associação de sulfametoxazol/trimetroprima e levofloxacino. No segundo caso, o paciente de 49 anos foi internado apresentando febre, náuseas, sudorese e tosse seca, também com suspeita de pneumonia. Das culturas de escarro, secreção traqueal e lavado bronco-alveolar, foi isolado BB, infecção tratada com ciprofloxacino: porém, o paciente foi a óbito.

**CONCLUSÃO:**

BB deve ser incluído na etiologia de pneumonia em pacientes imunocomprometidos com HIV. Pelo que é de nosso conhecimento, estes dois relatos foram os primeiros casos de pneumonia por BB que ocorreram neste hospital universitário.

## INTRODUCTION

*Bordetella bronchiseptica* (BB) is a strictly aerobic Gram-negative coccobacillus that causes diseases in the respiratory tract of animals, such as infectious tracheobronchitis or “canine cough” and pneumonitis in dogs, pneumonia in cats, coryza, pneumonia and otitis media in rabbits and bronchopneumonia in pigs.^1^^,^^2^^,^^3^^,^^4^^,^^5^

 Infections caused by BB are rare in humans but this bacterium can be isolated as a commensal agent in the human respiratory tract.^1^^,^^2^^,^^4^^,^^5^^,^^6^ BB occurs independently from contact with colonized animals, and can persist in the environment for long periods of time.^7^^,^^8^ In general, humans acquire BB through contact with domestic animals but this can also occur through cross-contamination with hospital patients.^1^^,^^2^

In human beings, BB preferentially attacks the respiratory tract and can cause severe pulmonary infections, such as infectious bronchitis, pneumonia and bacteremia. It generally occurs in patients with weak immune systems, such as AIDS patients.^6^^,^^7^^,^^8^^,^^9^

Two cases of pneumonia caused by BB in HIV-positive patients at a university hospital are reported below.

## CASE REPORTS

### Report 1

**Figure 1.**
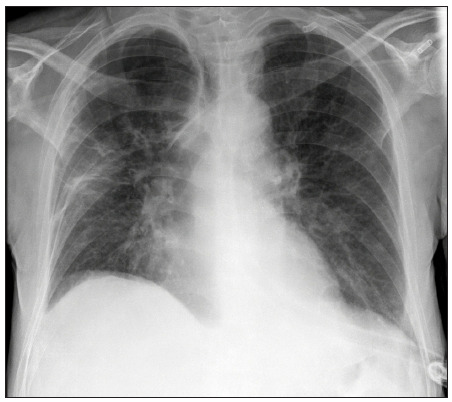
Lung radiograph of the patient in the first report, indicating pneumonia caused by *Bordetella bronchiseptica*.

The patient was a 43-year-old HIV-positive man who had been diagnosed with AIDS in 2006 and was positive for the hepatitis C virus (HCV). He was a homeless drug addict and alcoholic. He had pleural tuberculosis in 2000 and pulmonary tuberculosis in 2012. He was admitted to hospital in 2007 to investigate a cerebral mass lesion suggestive of toxoplasmosis or lymphoma. This diagnosis was not confirmed at the time but the patient showed some improvement with treatment for neurotoxoplasmosis. In November 2012, he was again admitted for treatment of nervous conditions caused by HIV/AIDS. In August 2013, the patient was admitted again showing deep vein thrombosis (DVT) and a subacute respiratory condition that indicated pneumonia, as shown on lung radiography (Figure 1). The treatment consisted of anticoagulant therapy and empirical treatment for pneumocystosis. Because the patient showed clinical improvement, he was released.

In May 2014, the patient was admitted again for treatment of disseminated AIDS-related disease, presenting a viral load of 589 copies/ml (reference value, RV: 493-1666 copies/ml) and CD8 212 cell/ml (RV: 224-1112 cell/ml). He complained of chronic pain in the legs, sacral eschar and a dry cough. During his stay in the hospital, antiretroviral treatment was started, with good tolerance to the medicine. Several tests were performed: routine blood tests, C-reactive protein and sputum and blood cultures.

The hemogram showed neutropenia compatible with bacterial infection; elevated C-reactive protein, 0.91 mg/dl (RV: below 0.3 mg/dl), thus suggesting inflammation or infection. BB was isolated from the sputum sample. At this point, antibiotic therapy with trimethoprim/sulfamethoxazole was started to combat BB. However, the antibiogram showed that the bacterium was sensitive to amoxicillin/clavulanic acid, piperacillin/tazobactam, meropenem, amikacin, gentamicin, nalidixic acid, levofloxacin, ciprofloxacin and norfloxacin; that it had an intermediate profile in relation to ampicillin, cefalotin and cefepime; and that it was resistant to cefuroxime, cefuroxime axetil, ceftriaxone, nitrofurantoin and trimethoprim/sulfamethoxazole. Thus, the antimicrobial therapy was changed to levofloxacin. The blood culture was negative.

The patient was released at the end of May 2014, with a recommendation to use HIV antiretroviral medication and the antimicrobial agent for an additional seven days.

### Report 2

**Figure 2.**
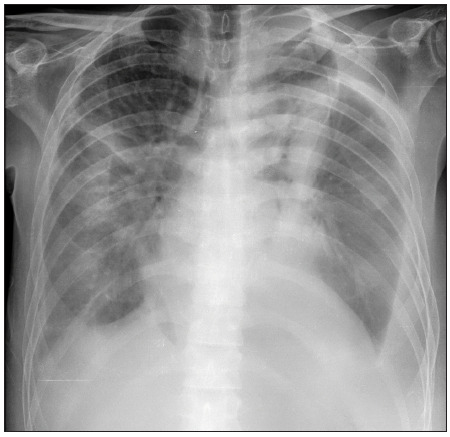
Lung radiograph from the second report, indicating pneumonia caused by *Bordetella bronchiseptica*.

The patient was a 49-year-old male who had been diagnosed positive for HIV, AIDS and HCV in 2012, with no treatment. He had been a smoker for 37 years. He presented hepatitis C with a heterogeneous mass lesion anterior to the 12^th^ thoracic vertebra. In April 2014, the patient reported that he had low-intensity lumbago irradiation pain for one month. Two weeks later, he reported intensified pain irradiating to the abdomen. A biopsy of the mass lesion was performed and showed a benign result. The patient was admitted to hospital at the end of May 2014 with a condition of night sweating, fever peaks (39 °C), abdominal pain, nausea, anorexia and pain in the left thigh for the last 30 days. He presented a viral load of 427 copies/ml, CD4 60 cell/ml (RV: 493-1666 cell/ml) and CD8 180 cell/ml (RV: 224-1112 cell/ml). He presented symptoms of peritoneal irritation. He had also been suffering from dyspnea and a dry cough for the last five days. He was suspected of having pneumonia, as shown on lung radiography (Figure 2). Therefore, empirical treatment for pneumonia was started.

At the end of May he was taken to the intensive care unit (ICU) because of complications.

**Figure 3.**
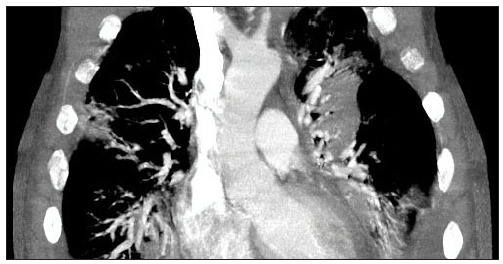
Thoracic computed tomography from the second report.

At the beginning of June 2014, the patient presented respiratory insufficiency. Thoracic computed tomography (CT) was performed, focusing especially on the right side (Figure 3). He remained in the ICU until June 10. Tests comprising sputum culturing, tracheal secretion culturing, bronchoalveolar lavage and blood culturing were then done. The blood cultures showed negative results, but BB was isolated from the samples of the other clinical specimens. The antibiogram of the three materials showed the same sensitivity profile: sensitivity to amikacin, ciprofloxacin, colistin, gentamicin, imipenem, meropenem, piperacillin/tazobactam, trimethoprim/sulfamethoxazole and tigecycline; intermediate profile towards ampicillin/sulbactam, ampicillin, cefepime and ceftazidime; and resistance to ceftriaxone and cefuroxime. The patient received treatment for BB initially with trimethoprim/sulfamethoxazole; this was then replaced by treatment with ciprofloxacin (10 days) and meropenem (7 days). At this point, the patient’s contact with 25 domestic dogs became known. The patient died at the end of June, and the possible causes included septicemia, pneumonia, AIDS and hypertension.

## DISCUSSION

We reported two cases of AIDS patients suffering from pneumonia due to BB. This microorganism is responsible for rare cases of chronic infection of the respiratory tract in humans, especially among individuals with underlying diseases.^10^ Formerly, BB used to be an uncommon cause of infection in hosts with a compromised immune system.^11^ However, cases among immunocompromised patients have been reported especially among those with HIV. The clinical condition is generally associated with infections in the upper respiratory tract, pneumonia, endocarditis and bacteremia.^6^^,^^9^^,^^12^ In our review of the literature using the Medline (http://www.ncbi.nlm.nih.gov/pubmed/), Embase (http://www.elsevier.com/online-tools/embase), SciELO (http://www.scielo.org/php /index.php) and Lilacs (http://lilacs.bvsalud.org/en/) databases, we did not find any article describing pneumonia in HIV-positive patients caused by *Bordetella bronchiseptica* (Table 1).

**Table 1.**
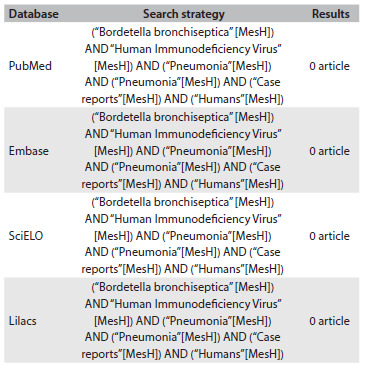
Review of medical databases using the descriptors corresponding to the main features presented by the patient, conducted on December 2, 2015

Since 1991, rare cases of BB have been reported in AIDS or immunocompromised patients.^9^^,^^13^^,^^14^^,^^15^ One of these reports was similar to the two reported above: a 42-year-old HIV-positive patient with pulmonary symptoms similar to pneumonia caused by *Pneumocystis jiroveci*. The sputum culture was positive for BB and treatment with levofloxacin, trimethoprim/sulfamethoxazole and azithromycin was successfully used. The patient had been in contact with a domestic dog.^9^

Wernli et al. described eight cases of BB that caused infection or colonization in human beings over a 15-year period.^16^ Those researchers showed that seven of their patients had underlying diseases and that only three had been in contact with domestic animals. The patients’ symptoms ranged from no symptoms to severe pneumonia. It was not possible to establish a homogeneous pattern regarding clinical disease among the symptomatic patients.^16^

A study by García-de-la-Fuente et al. in 2015 showed that most of the patients from whom BB was isolated presented a compromised immune system as well as an underlying disease, and 82% presented respiratory symptoms.^17^ Respiratory tract diseases are the major cause of morbidity and mortality among AIDS patients. The etiology of these respiratory infections varies according to factors such as CD4 levels, location of home, socioeconomic conditions and use of chemoprophylaxis. The CD4 count is the main risk predictor for progression to AIDS and death among HIV-positive patients.^18^

Although *B. bronchiseptica* is only rarely isolated in humans, it should be considered to be potentially pathogenic when found in samples from the respiratory tract in patients with a compromised immune system.^4^^,^^5^^,^^6^^,^^16^ Sputum culturing and investigation of exposure to animals are recommended.^9^^,^^16^ It became known that the patient in Report 2 had been in contact with 25 domestic dogs. These animals were the potential source of the acquired infection.^1^^,^^2^

Empirical antibiotic therapy using trimethoprim/sulfamethoxazole can be justified, given that this drug is the first choice for treating pulmonary pneumocystosis. This is caused by the unicellular fungus *Pneumocystis jiroveci*, a saprophyte of the respiratory tract that causes pneumonia in many HIV-positive patients. Its occurrence decreases substantially through antiretroviral therapy.^18^^,^^19^

Given that the antimicrobial treatment for infections caused by BB has not been clearly established, ascertaining the sensitivity profile of the antimicrobials is fundamental.^20^ García-de-la-Fuente et al. studied 36 respiratory tracts from which BB was isolated and found that the minimum inhibitory concentration (MIC) of antimicrobial agents was smaller for tigecycline, colistin, tetracycline, minocycline, doxycycline and meropenem; and it was larger for beta-lactams, macrolides and trimethoprim/sulfamethoxazole.^17^ According to other studies, this microorganism generally presents strong resistance to macrolides and clindamycin. Thus, treatment can be done using trimethoprim/sulfamethoxazole, fluoroquinolones and penicillins.^11^^,^^16^

Other studies have reported 100% sensitivity in vitro to aminoglycosides, penicillins and cephalosporins with anti-*Pseudomonas* activity, and to carbapenems, quinolones and tetracyclines.^6^ The Clinical and Laboratory Standards Institute (CLSI) does not recommend inclusion of erythromycin and clindamycin in the antibiogram; it advises that the antibiogram should be produced using quantitative methodology, including gentamicin, tobramycin, piperacillin and ceftazidime as the first line (group A); and piperacillin/tazobactam, ticarcillin/clavulanic acid, cefepime, amikacin, aztreonam, imipenem, meropenem, ciprofloxacin, levofloxacin and trimethoprim/sulfamethoxazole (group B) for the more resistant isolates.^21^

*B. bronchiseptica* has only rarely been isolated from clinical human specimens, in spite of the considerable exposure to this microorganism through colonized animals.^7^ Antimicrobial treatment should be started as soon as possible, especially in immunocompromised patients.^6^

## CONCLUSION

In spite of the fact that BB is associated with colonization in the respiratory tract and bronchopulmonary disease, it is difficult to clearly establish the pathogenic effect of this microorganism in human beings; its isolation presents challenging results. However, BB should be considered to be a possible agent for pneumonia since it has virulence factors, such as production of toxins. Isolation of BB from biological materials makes it possible to know its resistance profile, which can improve the prognosis considerably. As far as we know, up to the present time, the two cases reported above were the first cases in this university hospital.
